# Clinical Outcomes After Physical Therapy Treatment for Secondary Lymphedema After Breast Cancer

**DOI:** 10.7759/cureus.4779

**Published:** 2019-05-30

**Authors:** Cynthia Tan, Christopher M Wilson

**Affiliations:** 1 Physical Therapy, Beaumont Health, Royal Oak, USA; 2 Physical Therapy, Oakland University, Rochester, USA

**Keywords:** manual therapy, manual therapy, physical therapy, physical therapy, quality of life, quality of life, rehabilitation, exercise

## Abstract

Breast cancer is the most commonly diagnosed cancer in women and approximately 33% of survivors will develop lymphedema. Untreated lymphedema may be limb threatening or cause substantial functional limitations. The purpose of this case report is to detail the physical therapy (PT) management and outcomes for a patient with right upper extremity and breast lymphedema. The goal of this case report is to provide rehabilitation clinicians with an example of effective treatment management and the underlying treatment rationale. A 64-year-old female with stage 2A breast cancer underwent neoadjuvant chemotherapy, a lumpectomy with 18 lymph nodes removed, and radiation therapy. She subsequently developed secondary lymphedema of the right breast and upper extremity. Physical therapy interventions included instruction on a complete decongestive therapy program, which consists of manual lymphatic drainage and compression bandaging and exercises to improve shoulder range of motion (ROM), posture, and strength. As a result of PT, her right shoulder ROM and anthropometric measurements improved and the patient achieved independence with self-lymphatic massage and compression bandaging techniques to maintain these gains. This case report is unique as it details the clinical decision making required during a complex course of cancer care that necessitated adjustments to the PT plan of care for sustainable outcomes.

## Introduction

The American Cancer Society estimated in 2019 that over 271,270 women would receive a new diagnosis of breast cancer and it is the most common cancer affecting women in the United States. This accounts for 30% of all cancers in women [1]. It is estimated that 33% of breast cancer survivors will develop lymphedema within the first 6-18 months after treatment [2]. Upper extremity (UE) swelling may develop in weeks to years after breast cancer treatment and can cause dysfunction, pain and difficulty performing activities of daily living (ADL). In some cases, untreated lymphedema can cause infection and even be limb threatening. Lymphedema is characterized by an increase in protein-rich fluid within the interstitial space. This buildup occurs due to the interruption of the lymphatic system due to the surgical lymph node dissection, scar tissue or radiation fibrosis [3]. Lymphedema results when the lymphatic system cannot adequately remove protein from local tissues and manifests as fluid buildup. Lymphedema can present in the extremities, head and neck, trunk, or external genitalia [4].

Primary lymphedema is a developmental abnormality of the lymphatic system, which is either hereditary or congenital. There are various types of primary lymphedema, including aplasia, hypoplasia, hyperplasia, fibrosis of the lymph nodes, agenesis of lymph nodes, congenital, lymphedema praecox and lymphedema tarda. Secondary lymphedema is due to a mechanical insufficiency caused by a known insult to the lymphatic system [4]. Most common causes of secondary lymphedema include surgery, radiation, trauma, infection, malignant tumors, immobility and chronic venous insufficiency. In breast cancer survivors, the highest incidence of lymphedema occurs among those treated with breast surgery, axillary lymph node dissection, and radiation therapy [4].

Currently there is no cure or permanent remedy for lymphedema. If it is left untreated, lymphedema will gradually progress through its stages. Zuther and Norton [4] defined the stages of lymphedema as: “The latency stage is characterized as having no swelling. Stage 0, or subclinical stage, is characterized as having a reduced transport capacity (TC) and normal tissue consistency. Stage 1, the reversible stage, is characterized as having soft, pitting edema, no secondary tissue changes, and elevation can assist in reducing the swelling. Stage 2, spontaneously irreversible stage, is characterized as having lymphostatic fibrosis, hardening of the tissue, no pitting, positive Stemmer sign, and having frequent infections. Stage 3, also known as lymphostatic elephantiasis, is characterized as extreme increase in volume and tissue texture with typical skin changes (papillomas, deep skinfolds, etc.) and positive Stemmer sign.”

Lymphedema swelling may cause discomfort and sometimes disability. It can lead to cellulitis and lymphangitis, predisposing the patient to systemic and sometimes life-threatening infection if left untreated [5]. The physical and psychological aspects of the condition greatly impact the daily lives of those diagnosed with lymphedema [6]. In addition to having surgery, risk factors have been identified that increase the likelihood of development of secondary lymphedema, including elevated body mass index (BMI), type of surgery, infection and injury [7]. The purpose of this case report is to describe the initial findings and the physical therapy management for a patient presenting with upper extremity deficits and lymphedema after breast cancer treatments. This case was completed according to CARE Guidelines for reporting clinical case reports [8].

## Case presentation

During a routine mammogram, a 64-year-old female was found to have two 7-mm focal asymmetries and lymphadenopathy. Her initial cancer stage was diagnosed as cT1cN1cM0. Four weeks after her positive mammogram, she underwent a needle biopsy of an axillary lymph node that demonstrated malignancy. She was initially diagnosed with right breast cancer stage 2A which was ER+, PR+ and HER-2Neu negative. The surgical biopsy was then diagnosed as metastatic invasive lobular carcinoma. Upon pathological staging she was subsequently staged as Stage 3C. Her final stage via pathology was pT1N3M0. Her past medical history included hypertension, endometriosis and a benign thyroid nodule. Her surgical history included a hysterectomy and a hernia repair of unknown site. She was a retired social worker and a widow.

After initial staging, the patient had neoadjuvant chemotherapy. She completed four out of four cycles of cyclophosphamide with doxorubicin and subsequently completed eight out of eight cycles of taxol. She responded well to chemotherapy with no significant treatment delays or major side effects. After completion of chemotherapy, she underwent a right lumpectomy with axillary lymph node dissection. Fifteen out of 18 dissected lymph nodes were positive for malignancy. Three weeks after surgery, a bone scan showed no evidence of skeletal metastases. Her lumpectomy margins were also found to be inadequate which required a re-excision of the right breast four weeks after her initial lumpectomy.

She received an initial episode of physical therapy (PT) for right upper extremity (UE) lymphedema for eight visits by another clinician. This retrospective chart review resulted in limited availability of data and clinical insights into the patient's initial episode of care therefore we will focus on the second episode of care. The patient had initially presented with minimal swelling of the UE and moderate swelling of the right breast and chest wall. She also demonstrated decreased right shoulder range of motion (ROM) for flexion and abduction which resulted in difficulty with raising her arm overhead as well as pushing and pulling. After the eight visits of PT, she required minimal assistance with compression wrapping and swelling had decreased by approximately 1 cm. Her right shoulder flexion had improved to 150° and abduction to 142°. Her therapy was discontinued early due to a new finding of a renal mass that required a nephrectomy.

As a component of her routine follow-up, she underwent a computerized tomography scan that found a right kidney mass that was suspicious of carcinoma. A biopsy revealed papillary renal cell carcinoma which required a right radical nephrectomy. Concurrently, her radiation oncologist recommended external beam radiation to her breast and axilla. She was also started on anastrozole (Arimidex). Her radiation therapy was delayed one month due to the nephrectomy. She completed 30 cycles of external beam radiation over six weeks. Throughout her radiation treatments, she received ongoing consultations and screening by a physical therapist who was stationed in the radiation oncology department.

Clinical findings

**Figure 1:**
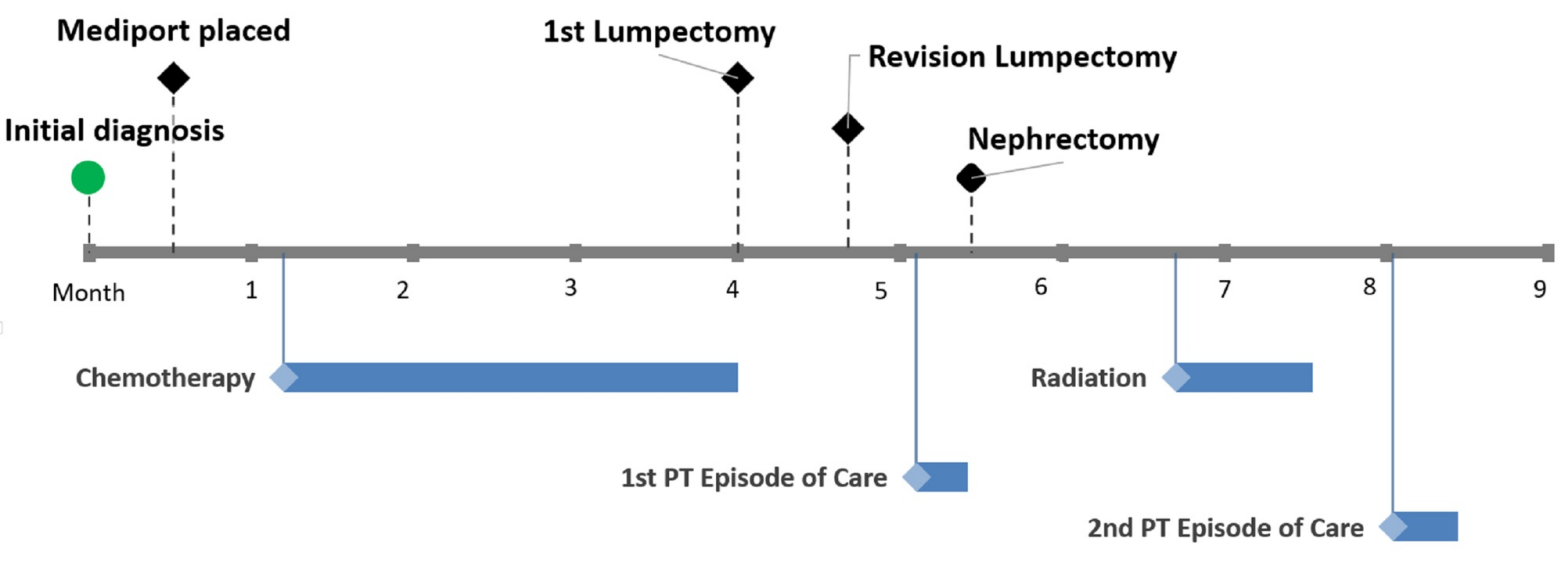
Cancer Treatment Timeline Black diamond = surgical treatment/procedure; blue diamond/bar = episode of treatment and duration

The case described was the patient’s second episode of care as the first episode of care was interrupted by the need for a radical nephrectomy. This second episode of care lasted for 10 visits and the patient was seen three times per week. This episode of care was initiated 50 weeks after her initial mammogram, 26 weeks after her lumpectomy and 17 weeks after discharge from her first episode of outpatient PT (Figure 1). All measurements and examination procedures were completed by the same physical therapist for this episode of care.

Subjective History

Upon listening to the patient’s subjective remarks regarding when the swelling started and how it began post-surgically, surgical swelling was on the initial differential diagnosis list. However, as the swelling remained longer than two to three weeks and there was not a clear inflammatory process, lymphedema was considered to be more likely. In addition, the likelihood of lymphedema was increased due to her history of radiation and a BMI greater than 30. She also reported difficulty with reaching above her head as well as tightness and a pulling sensation when reaching above or behind her; this would require examination for lymphatic cording after lymph node impairment. The examination also included assessing for infection or cellulitis which may precipitate lymphedema. Prior to the initial history, the patient completed an Upper Extremity Functional Index (UEFI), in which the patient reported difficulty reaching into high cupboards, putting a shirt on over her head and lifting a bag of groceries. The patient’s overall UEFI score was 48/80 with a score of 80 being no difficulty with any tasks (Table 1). The patient’s goals were to decrease her pain, size of her right breast and upper extremity and improve her shoulder ROM in order to be independent with home maintenance activities and performance of her ADLs. Based upon her prior episode of PT care for her right UE lymphedema, it was anticipated that she would be knowledgeable of her lymphedema diagnosis and management, however upon the initial subjective history, it was found that this foundational knowledge was lacking.

**Table 1:** Upper Extremity Functional Index Scores for 2nd Episode of Care Total score out of 80 (0 = most severe limitation; 80 = least limitation).

	Initial Evaluation	Discharge Evaluation
Total Score	48	66
Extreme Difficulty or Unable to Perform Activity (0)	0	0
Quite a Bit of Difficulty (1)	3	0
Moderate Difficulty (2)	8	2
A Little Bit of Difficulty (3)	21	36
No Difficulty (4)	16	28

Tests and Measures

Postural and palpatory assessment: Upon observation of the patient’s physical stature, she was moderately overweight, with obvious difference in size of the upper extremities and breasts. She demonstrated postural deviations including a slight forward head, rounded shoulders, and a posterior pelvic tilt. She also presented with a concave chest wall. Her right breast was observed to be larger and had a peau d’orange type texture along the inferior border of the right breast with an increase in fibrotic tissue of the right breast. She was also more sensitive to palpation of the right breast, especially along the lateral and inferior side of the breast. She demonstrated increased palpatory tenderness of the posterior aspect of the right axilla which is consistent with the symptom presentation pattern for lymphatic cording. There were no signs of cellulitis, infection or other inflammatory process.

Objective measurements: Upper extremity and trunk girth measurements were taken utilizing a standard cloth tape measure (Tables 2, 3). Diagnostic criteria for lymphedema is generally accepted to be a 10-15% increase in total extremity volume and may be reflected by a 2-cm increase in anthropometric measurement as assessed via tape measure [9]. Standard landmarks were utilized to compare repeated measures. The patient's shoulder ROM was assessed via a plastic goniometer in supine. The strength of her bilateral shoulders was quantified via manual muscle testing (MMT) in sitting as described by Kendall (Table 4). Her cervical spine range of motion was unremarkable.

**Table 2:** Circumferential Anthropometric Measurements of the Upper Extremities All measurements taken in supine and in centimeters (cm).

Landmark Distance from Tip of 3^rd^ Digit	Left (unaffected)	Right Initial Evaluation	Right Discharge Evaluation
10	18.1	17.9	17.2
18	15.5	16.1	14.9
28	22.2	22.9	21.3
38	26.6	28.9	25.4
48	31.7	34.2	30.7
58	33.9	35.6	32.8

**Table 3:** Circumferential Anthropometric Measurements of the Breast and Trunk All measurements in centimeters (cm). All measurements taken in standing with shoulders at 90° of abduction and at the end of exhalation.

Landmarks for Circumferential Measurements	Initial Evaluation	Discharge Evaluation
Axilla	92.6	89.3
10 cm below axilla	95.3	91.5
20 cm below axilla	104.6	99.1

**Table 4:** Shoulder Objective Measurements Left shoulder active range of motion was unremarkable. AROM = Active Range of Motion; MMT = Manual Muscle Test

	Initial Evaluation	Discharge Evaluation
Right Shoulder Flexion AROM	129°	147°
Right Shoulder Abduction AROM	124°	146°
Right Shoulder Flexion Strength (MMT)	3+/5	4+/5
Left Shoulder Flexion Strength (MMT)	4/5	5/5
Right Shoulder Abduction Strength (MMT)	3+/5	4+/5
Left Shoulder Abduction Strength (MMT)	4/5	5-/5

Physical Therapy Diagnosis

The patient was classified with Stage 2 secondary lymphedema without significant complications. Stage 2, also called spontaneously irreversible stage, is characterized as having lymphostatic fibrosis, hardening of the tissue, no pitting, positive Stemmer sign, and increased risk of frequent infections [4]. As this lymphedema was precipitated after a cancer diagnosis and disruption of lymphatic pathways due to surgery and radiation therapy, it was anticipated that this would become a chronic condition.

Evaluation and Clinical Decision Making

The likelihood of lymphedema was increased due to the history of lymph node dissection, radiation, and surgery. This clinical suspicion was further reinforced by the objective findings of increased anthropometric measurements and skin changes of her breast and upper extremities as well as her AROM and strength deficits. Based on these findings, the patient would benefit from education for a home exercise program (HEP) including stretches, strengthening exercises and patient education on self-lymphatic massage. The most important issues were to focus on achieving the patient’s goals of improving her function by reducing swelling of the right upper extremity and breast, as well as educating her on how to manage the diagnosis independently. This would also include instructing her on a HEP to improve her posture, shoulder AROM and strength.

Based on observational assessment of her sitting and standing posture, she would benefit from exercises to assist in achieving and maintaining optimal upright posture which included chin tucks and scapular retraction. In addition, the patient would require education on the lymphatic system and the components of comprehensive decongestive therapy (CDT). The tightness of right pectoralis muscle was believed to be contributing to her shoulder ROM limitations, therefore, instruction on postural stretches, including a pectoral doorway stretch and use of a wand to do shoulder flexion and abduction stretches, would be required. Additionally, the patient would be educated and coached on aerobic exercise via a walking program to improve cardiovascular fitness. As an adjunct to her physical therapy, she was encouraged to utilize the facility’s indoor track and instructed to do simple stretches and strengthening exercises with both elastic bands and light weights. The patient received education on the health system’s Cancer Survivorship Exercise and Wellness aftercare program where she could continue with an exercise program after she was discharged from physical therapy to continue to progress her strength and cardiovascular endurance and maintain her ROM. In order to evaluate the patient's ongoing progress, at every treatment the patient would be asked how long she wore her compression padding for her breast swelling and the compression bandaging for her right upper extremity and whether she had been compliant with her self-lymphatic massage and HEP.

Therapeutic interventions

There were several goals that were pursued in physical therapy: decreasing anthropometric measurements of the right upper extremity and breast, instructing the patient on self-lymphatic massage and compression bandaging, and achieving independence in a HEP to improve her shoulder AROM, strength and posture. Physical therapy started with manual lymphatic drainage, education on her lymphatic system and the rationale for its dysfunction. To reduce the swelling of the right upper extremity and breast, compression bandaging of the right upper extremity and breast was also initiated. On treatment day three, the patient was instructed in proper performance of self-lymphatic massage and compression bandaging. In order to assure steady limb and breast reduction, she would be required to perform these tasks at home on the days she was not in PT. Every treatment session after day three, the patient and therapist engaged in a thorough review of self-lymphatic massage and compression bandaging until she was able to consistently provide return demonstration for accurate performance of these techniques. Once the patient's breast edema was reduced, she was advised to wear a compression bra to maintain this reduction.

To decrease the patient's stress and anxiety related to incorporating the lymphedema program into her current lifestyle, the patient received extensive education on the clinical rationale for the various treatments and the patient was provided an opportunity to ask clarifying questions throughout each treatment. Education also included instruction to remove the compression bandages in the event of sharp pain, numbness or tingling. Finally, the patient received a handout and a personalized video recording of the self-lymphatic massage and compression bandaging to assist with compliance and performance.

The patient received manual therapy including trigger point releases of the right upper quadrant to reduce tightness of the pectorals, subscapularis, teres minor and major, trapezius, infraspinatus, latissimus dorsi and rhomboids to help increase shoulder ROM and assist in achieving upright posture. She was also given active upper extremity exercises to perform while wearing her compression bandages. These exercises were aimed to assist in actively pumping the fluid out of the right upper extremity and included elbow flexion/extension, wrist flexion/extension and fist clenches. These active exercises would use the compression bandaging as a counterforce to pump the fluid out of the upper extremity to assist in decreasing her anthropometric measurements.

Key postural exercises provided included chin tucks, shoulder shrugs, shoulder circles, and scapular retraction/adduction. The patient received shoulder ROM exercises, including shoulder flexion and abduction with a wand, and supine gravity-assisted shoulder flexion. As the patient progressed, she also received new stretching exercises to increase her shoulder ROM and postural stretching and strengthening exercises to help the patient sustain an upright posture. The initial shoulder stretching regimen included passive shoulder flexion on a table, shoulder abduction with a wand to improve glenohumeral and scapulothoracic motion. She also performed shoulder extension and adduction with elastic bands that provided moderate resistance. She also performed shoulder flexion and abduction exercises with light weight and high repetitions. She also received exercises in a supine position that she could perform on her bed at home. These included alternate shoulder flexion stretches and shoulder abduction to increase pectoral ROM. Finally, she was given strengthening exercises in supine including shoulder flexion with elastic bands from 90° flexion to her end range, horizontal shoulder abduction/adduction and internal/external rotation with the arm at the side of the body with elastic bands. In standing, she was instructed in strengthening exercises including shoulder extension and adduction with an elastic exercise band and shoulder flexion and abduction exercises and biceps curls and triceps extension using light weights with approximately two sets of 15 repetitions.

Outcomes

Physical therapy appeared to be effective as the patient demonstrated a decrease in anthropometric measurements of the upper extremity and trunk (Tables 2, 3). Tidhar et al. reported a minimally clinically important difference of circumferential measurements as greater than 1% for either the arm or leg [10]. As the standard error of measurement (SEM) for anthropometric measurements was less than 1%, the patient demonstrated a greater than 1% reduction in swelling for all measurements.

There was an increase in shoulder ROM for abduction of 22° (Table 4). A reduction in shoulder abduction ROM of ≥20 degrees is associated with greater difficulty with household activities and hobbies [11]. Using the UEFI, it was observed that the patient had an improvement in her UE function and ADLs since beginning physical therapy (Table 1). This change was found to be greater than the minimal detectable change of 7.9 scale points as cited by Binkley et al. [12].

As the patient was able to achieve her initial goals of becoming independent with the self-lymphatic massage and the self-compression bandaging and decreasing her anthropometric measurements, the focus of therapy shifted toward other goals. These goals included addressing her posture, shoulder ROM and strengthening exercises. Physical therapy helped this patient take control over her physical issues by decreasing the swelling of the right UE and breast. The patient’s improvement in her shoulder ROM now allowed her to be able to reach into higher cupboards with less difficulty. The education on self-lymphatic massage and compression bandaging empowered the patient to manage this condition herself if she encountered a re-exacerbation in swelling.

After discharge, the patient was advised to continue with self-lymphatic massage and compression bandaging at night for the next three months on a daily basis to maintain the decreased anthropometric measurements and wear her compression sleeve and compression bra throughout the day. At the three-month mark, she was instructed to start weaning herself off the compression bandaging and to schedule a follow-up with her physician. The therapist also recommended utilizing the unaffected arm for blood pressure measurements and blood draws/punctures to reduce the risk of exacerbation of lymphedema. She was also instructed not to submerge herself in a hot tub or sauna due to increasing her chance of exacerbating her lymphedema. She was given handouts on skin care and helpful guidelines. The patient was encouraged to increase her daily walking program to 30 minutes and continue with the stretching and strengthening home exercises.

## Discussion

As a key component of the overall treatment approach, the patient experienced a clinically significant decrease in anthropometric measurements and fibrotic tissue of the upper extremity and breast after CDT. Physical therapy including CDT has been shown to reduce limb volumes in patients at risk of progressive lymphedema [13]. A systematic review of lymphedema management by Armer et al. found that lymphedema is effectively managed by a holistic plan that includes manual lymph drainage [14]. This patient had a positive experience with this round of physical therapy due to incorporating CDT, therapeutic exercises and neuromuscular re-education to continue educating the patient on ROM, postural and strengthening exercises that can be done easily at home for continued compliance after being discharged. Breast cancer survivors have been known to have impaired shoulder mobility and pain ranging from 8% and 45% approximately three months after surgery [14]. DeVoogdt et al. observed that with physical therapy, shoulder ROM and strength improved as well as activities of daily living [15]. Harrington et al. stated that patients will have deficits following breast cancer treatment in shoulder ROM and strength which need to be addressed with physical therapy [16]. In this patient’s case, she initially had shoulder limitations, but at discharge, the patient's shoulder ROM was within functional limits after having manual therapy and instruction on proper posture and strengthening exercises.

In comparing the clinical outcomes of this case report to a systematic review by Hasenoehrl et al., our case report demonstrated similar clinical outcomes to their findings that breast cancer-related lymphedema is well manageable and safely treated with complete decongestive therapy and resistance exercises [17]. Hasenoehrl et al. described substantial challenges during their systematic review of the literature as there were only 23 articles that could be identified on this topic and there was wide variation in research approaches that limited a thorough meta-analysis. Although not a clinical trial, our case report further contributes to the literature in order to assist in establishing predictable clinical outcomes in this patient population.

A limitation of this treatment approach was that financial barriers limited the longitudinal management of her case by the therapist. If there was no limitation to the amount of therapy, another goal that physical therapy should address would include cancer-related fatigue. The incidence of cancer-related fatigue is a common side effect and is reported to occur with 70-90% of cancer survivors [18]. Exercise has proven to be an effective treatment to reduce fatigue and improve quality of life [19]. Another limitation of this plan of care was that the patient was not seen for follow-up assessments after the conclusion of her second episode of care. These reassessments would allow the physical therapist to determine if the patient was maintaining her smaller anthropometric measurements and her shoulder ROM and strength. If she was having difficulties at the time of follow-up, the physical therapist could recommend another episode of care. If the patient was doing well, the physical therapist could progress her home exercise program. This approach is supported by Stout et al. but was challenging in the clinical practice environment due to scheduling and financial constraints [20].

## Conclusions

After breast cancer treatment, this patient developed secondary lymphedema, upper extremity weakness, and decreased shoulder range of motion and impaired activities of daily living and function. These side effects required two episodes of care of physical therapy which resulted in clinically significant improvements in anthropometric measurements, range of motion, strength and improvement in activities of daily living. She demonstrated independence in self-management of her lymphedema and improved in her Upper Extremity Functional index from 48/80 to 66/80. Early identification and referral to physical therapy to proactively address these issues may have decreased the risk of long-term side effects and the resulting healthcare services.
